# Anomalous Phonon Softening with Inherent Strain in Wrinkled Monolayer WSe_2_

**DOI:** 10.1002/adma.202419414

**Published:** 2025-04-10

**Authors:** Dong Hyeon Kim, Jaekak Yoo, Hyeong Chan Suh, Yo Seob Won, Sung Hyuk Kim, Dong‐Joon Yi, Byeong Geun Jeong, Chanwoo Lee, Dongki Lee, Ki Kang Kim, Seung Mi Lee, Eui Kwan Koh, Mun Seok Jeong

**Affiliations:** ^1^ Department of Physics Hanyang University (HYU) Seoul 04763 Republic of Korea; ^2^ Department of Energy Science Sungkyunkwan University (SKKU) Suwon 16419 Republic of Korea; ^3^ Department of Electronic Engineering Hanyang University (HYU) Seoul 04763 Republic of Korea; ^4^ Department of Nanotechnology and Advanced Materials Engineering Sejong University (SJU) Seoul 05006 Republic of Korea; ^5^ Korea Research Institute of Standards and Science Daejeon 34113 Republic of Korea; ^6^ Korea Basic Science Institute Seoul 02855 Republic of Korea

**Keywords:** inherent strain, phonon softening, raman selection rule, tip‐enhanced Raman spectroscopy (TERS), tungsten diselenide (WSe_2_)

## Abstract

Local deformation is a control knob to dynamically tune the electronic band structure of 2D semiconductors. This study demonstrates the local strain‐dependent phonon properties of monolayer tungsten diselenide, which are investigated by using the scanning tunneling microscopy‐based tip‐enhanced Raman spectroscopy. The anomalous appearance and softening of the Raman‐inactive out‐of‐plane 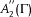 mode are first revealed, which exhibits equivalent behavior to other principal phonons of tungsten diselenide. Local strain calculations unveiled the linear proportionalities of 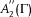 phonon nature on strain and it facilitates the derivation of Grüneisen parameter by experimental and theoretical approaches. Additionally, the origins of the anomalous appearance of the Raman‐inactive 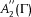 mode are clearly proved in both classical physics and quantum mechanics. Especially quantum mechanical calculations have precisely described strain‐induced selection rule relaxation by polarizability changes. The first discovery provides a fundamental understanding of the strain‐dependent phonon properties, as well as suggesting a new distinct strain indicator, 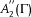 mode, for strain engineering.

## Graphical abstract

This study introduces the first discovery of a new local strain indicator,  mode, for monolayer WSe_2_‐based excitonic applications including single photon emitter by using tip‐enhanced Raman spectroscopy. The correlative investigation with density functional perturbation theory provides the origin of the anomalous appearance of  mode, as well as the applicability for strain engineering.

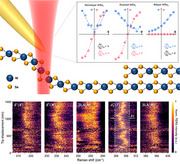

## 1 Introduction

Structural perturbations induced by intrinsic and extrinsic strain in the atomic lattice of transition metal dichalcogenides (TMD) facilitate modulation of the electronic band structure and localization of electronic potentials.^[^ ^1^ ^]^ These deformations can serve as funnels and localizers for quasiparticles like excitons, phonons, and polaritons, and they play a key role in the application of tungsten diselenide (WSe_2_) as efficient energy‐harvesting and optomechanical devices by controlling localized excitons in nanoscale.^[^ ^2^ ^]^ In particular, strained monolayer WSe_2_ shows promise as a single‐photon emitter (SPE) and quantum light source for quantum information technology, as it detunes dark excitonic state and interacts with defect states induced by atomic vacancies.^[^ ^3^ ^]^ These outstanding properties have spurred numerous researchers to fabricate monolayer WSe_2_‐based SPE using local strain applications such as nanoparticles, periodic nanostructured substrates, and nanoindentation by atomic force microscope (AFM).^[^ ^4^ ^]^

Recently, to effectively utilize lattice deformations in WSe_2_ for strain engineering, fundamental understandings of quasiparticles under diverse strains have been widely researched. Strain‐dependent spatial behaviors and temporal coherence of excitons are investigated by *g* ^2^ measurements, Hong‐Ou‐Mandel interference measurements, confocal‐, and nano‐photoluminescence microscopy.^[^ ^3^, ^5^ ^]^ Similarly, because lattice vibrations are directly influenced by internal and external strain, confocal Raman microscopy, polarized Raman spectroscopy, and surface‐enhanced Raman spectroscopy can reveal the precise degree of deformation, phonon‐induced dephasing, and electron‐phonon scattering.^[^ ^6^ ^]^ Despite the significance of phonon behaviors, it is challenging to probe the strain‐dependent phonon properties of 2D semiconductors at nanoscale areas due to the small cross‐sections of Raman scattered light and the low spatial resolution of conventional phonon analytic methods. To surpass those spatial and spectral limits, tip‐enhanced Raman spectroscopy (TERS) has been implemented to achieve strong signal enhancement in atomic‐scale resolution. Taking these analytical advantages into account, the application of TERS has rapidly increased in a short period to analyze nanoscopic properties such as low‐dimensional defect analysis, vibrational analysis of single molecules, visualizing quasiparticle distributions, and in‐situ investigation of molecular reactions.^[^ ^7^ ^]^

In this study, we carried out scanning tunneling microscopy (STM)‐based TERS to investigate the local strain‐dependent phonon properties in the nanoscale regime. The directly grown monolayer WSe_2_ on an atomic sawtooth Au substrate presents an inherent strain that varies with diverse curvatures. Here, we first demonstrated the anomalous appearance and softening of the forbidden out‐of‐plane vibrational 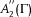 mode in wrinkled monolayer WSe_2_, due to the gigantic field enhancement, high spatial resolution of TERS, and strain‐induced relaxation of the Raman selection rule. According to our analysis, diverse modes, including the 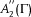 mode, exhibit a collective linear dependence of frequency change with local strain. Furthermore, we theoretically calculated the trends in phonon dispersion curves, polarizability, and dipole moment to perform a comparative analysis of local strain‐dependent phonon properties and to study the principles of selection rule relaxation in both classical physics and quantum mechanics. We anticipate that our new findings could be regarded as a milestone for strain engineering of monolayer WSe_2_.

## 2 Results and Discussion

### 2.1 STM and TERS Characterization of Wrinkled Monolayer WSe_2_ with Inherent Strain

**Figure 1:**
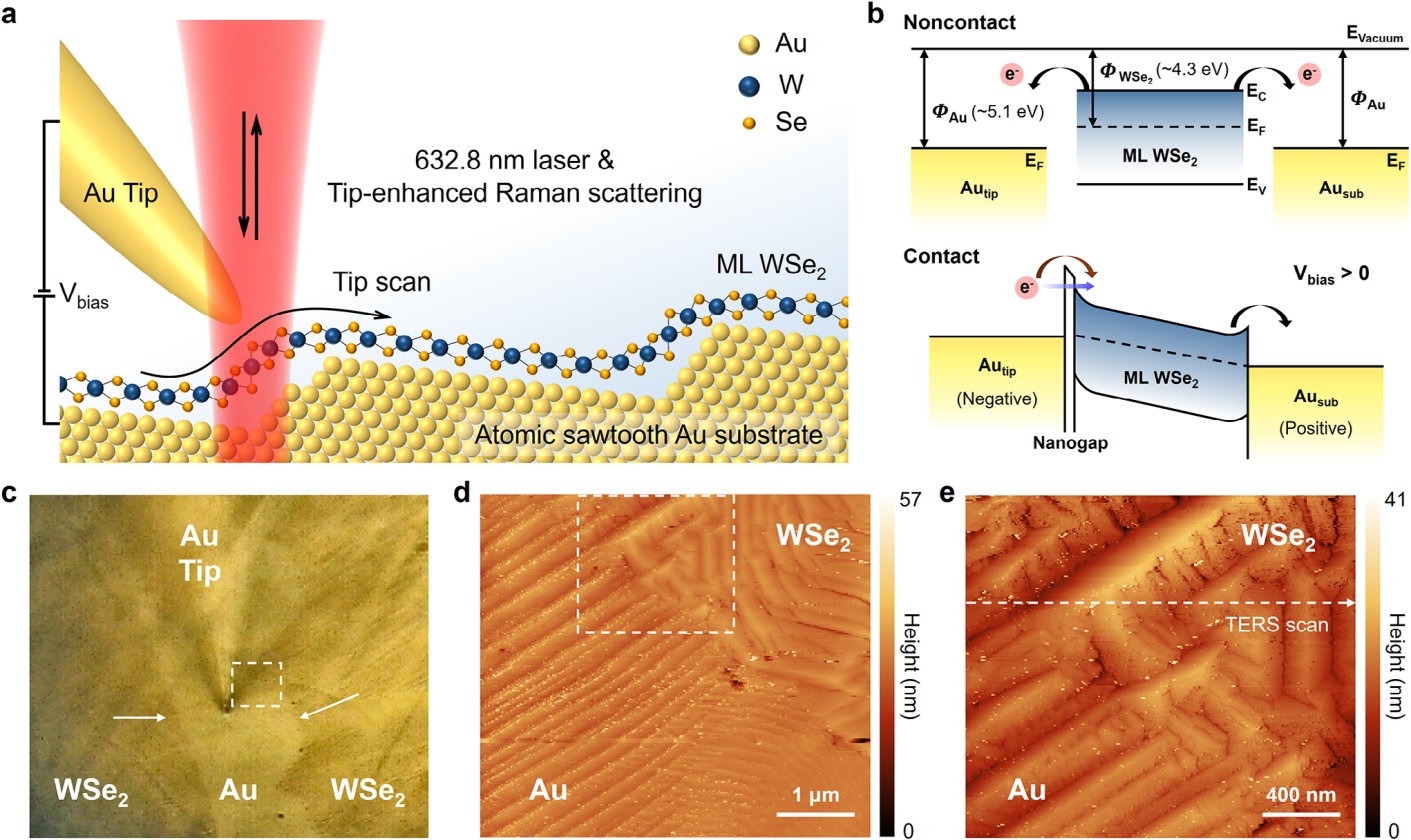
STM‐TERS measurement for the wrinkled monolayer WSe_2_. a) Schematic illustration of the TERS measurement. b) Energy band diagram of Au and WSe_2_ monolayer (ML) with the work functions (Φ) and the Fermi levels for the case with noncontact (top) and contact with applied bias voltage (bottom). c) Optical microscope image of sample. The white arrows indicate the boundary between the Au substrate and monolayer WSe_2_. d,e) Scanning tunneling microscope images of the sample with wrinkled structure. The white dashed rectangles in (c,d) indicate the scanning area of (d,e), respectively. The white dashed arrow in (e) indicates the TERS scan direction for the line trace measurement.

**Figure** **1**a illustrates the overall configuration of STM‐based TERS for the study of local strain‐dependent phonon properties at the nanoscale. We have utilized monolayer WSe₂ grown directly on an atomic sawtooth Au substrate, besides transferring it using various methods.^[^ ^8^ ^]^ This approach enabled us to investigate the inherent strain resulting from the direct contact and strong interaction between the Au atoms of the substrate and the Se atoms of WSe₂.^[^ ^9^ ^]^ The Au substrate not only served as the platform for the synthesis but also as the metal substrate for conducting STM and TERS measurements. Additionally, the direct contact without insulating layers facilitated stable STM measurements, which can give us precise STM images and induce photoluminescence (PL) quenching for clear analyses of Raman spectra under applied bias voltage, as shown in Figure 1b.

Initially, we measured STM topography over a wide area to briefly investigate the gold substrate and the wrinkle of the monolayer WSe_2_ which could present inherent strain. The scanning area is marked with a white dashed square in the optical microscope image, which includes an edge site between the monolayer WSe_2_ and the gold substrate, as indicated by the white arrow in Figure 1c. The interface between the sample and substrate is clearly distinguished by color and contrast differences (Figure S1, Supporting Information). Figure 1d shows the STM topography of the wrinkled WSe_2_ on the atomic sawtooth Au substrate, and Figure 1e is an enlarged STM image of the white dashed area in Figure 1d. The scanning electron microscope (SEM) image in Figure S2 (Supporting Information) further confirms the structure of the Au substrates observed in the STM measurements with obvious consistency.

**Figure 2:**
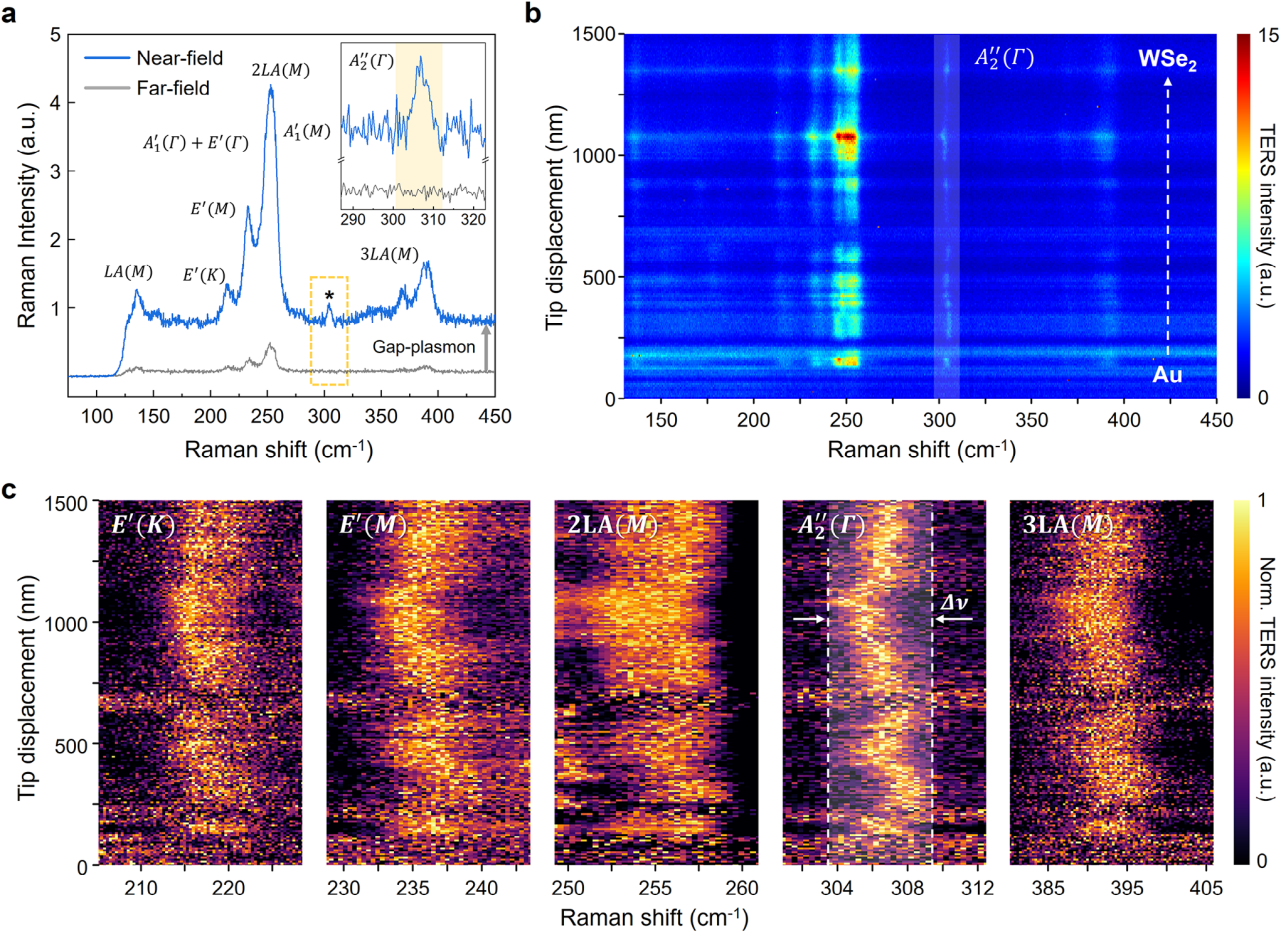
Tip‐enhanced Raman spectra of wrinkled monolayer WSe_2_. a) Representative near‐field and far‐field Raman spectra of sample. The inset is the magnified spectra of yellow dashed rectangle region in (a). b) TERS multispectral line trace data along the white dashed arrow in Figure 1e. The tip moves forward to the interior of monolayer WSe_2_ from Au substrate. The white area near 300 cm^−1^ emphasizes the distinct frequency shift of 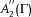 mode. c) Normalized TERS spectra of representative phonon modes along the tip displacement. These phonons show collective behaviors as the gold tip moves toward the wrinkles.

**Figure** **2**a shows representative far‐field and near‐field Raman spectra from wrinkled WSe_2_. As shown in the figure, most phonon modes in the near‐field Raman spectrum exhibit significant enhancement in signal intensity due to the gap‐plasmon as the TERS nanoprobe approaches. In addition to the increased signal intensity, various forbidden modes are excited by the strong gap‐plasmon resonance and perturbation of electromagnetic fields. Among these phonon modes the 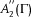 mode, located near 310 cm^−1^, appears anomalously. This phonon mode is widely known as the $B_{2g}^{1}$ mode in bulk TMD crystal, including WSe_2_. Based on point group theory, the 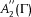 mode in monolayer TMD crystal is basically inactive in the Raman scattering process and usually allowed for IR absorption process.^[^ ^10^ ^]^ Moreover, this $B_{2g}^{1}$ vibrational mode becomes Raman‐active in bilayer TMD, where it is referred to as the $A_{1g}^{1}$ mode. By using the presence or absence of the 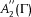 mode, it is possible to easily distinguish between monolayer and bilayer of WSe_2_. These characteristics are confirmed by the far‐field Raman spectrum inset in Figure 2a and Figure S4 (Supporting Information), which shows no phonon mode in the range from 290 to 320 cm^−1^.

Note that, there is extensive research that deals with the strain‐dependent phonon properties of TMD materials including synthesized/exfoliated monolayers, bulk crystals, nanoplatelets, nanosheets, and heterobilayer through experimental and theoretical investigations. Especially, to apply strain on TMDs, various methodologies such as bending samples through two, three, and four‐point clamps, in‐plane stretching the flexible substrates, and forming microstructure are utilized.^[^ ^3^, ^6^, ^11^ ^]^ However, the 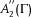 mode has not been observed in strain‐dependent confocal Raman microscopy studies of monolayer WSe_2_. Furthermore, the anomalous appearances of this phonon mode in monolayer WSe_2_ have been rarely observed under specific extreme conditions, such as strong field enhancement by metal nanoparticles and resonance Raman scattering process, but without detailed analysis of phonon properties due to the low spatial and spectral resolution.^[^ ^5^, ^10^ ^]^ To further investigate the local strain‐dependent properties of the 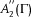 mode and other phonon modes at the nanoscale, we conducted TERS line trace measurement along the white dashed arrow in Figure 1e with 7 nm step from the Au substrate to the interior of the wrinkled WSe_2_. Figure 2b presents the TERS spectra from multispectral imaging acquired through line trace measurement. As shown in the figure, as the TERS nanoprobe moves toward the interior of the sample, the 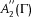 phonon mode exhibits not only changes in Raman signal intensity but also large frequency shifts, which have not been reported or studied before. Similarly, in Figure 2c, the 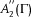 phonon mode shows a clear trend of frequency shifts along with tip displacement in the normalized TERS spectra, alongside other phonon modes such as *E*′(*K*), *E*′(*M*), 2LA(*M*), and 3LA(*M*). Interestingly, all these phonon modes demonstrate collective behavior in their frequency changes as the tip moves further into the sample. It is reasonable to consider that the change in tip displacement correlates with a change in inherent strain, given the wrinkled structure of the monolayer WSe_2_. Furthermore, it is appropriate to assume that the phonon frequency shifts are induced by local strain, with minimal influence from electron doping, as the applied voltage bias maintains a locally charge‐neutral state.

### 2.2 Local Strain‐Dependent Phonon Properties

**Figure 3:**
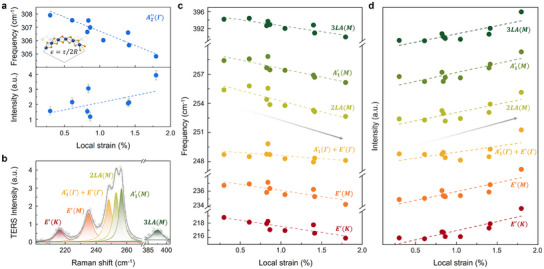
Linear relationship between vibrational properties and local strain. a) Linear dependence of 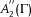 mode on local strain. 𝜖, τ, and 𝑅 are denoted as local strain, material thickness, and radius of curvature, respectively. b) Representative deconvolutions result of TERS spectra of monolayer WSe_2_ (ε = 1.4%). The grey empty circles and solid lines indicate the raw Raman spectrum and fit curves, respectively. c,d) Linear dependence of the frequency and intensity of principal Raman modes of monolayer WSe_2_ on local strain. The frequency and intensity value of various Raman modes were acquired by deconvolution processes.

While the TERS tip explores the sample to acquire the TERS spectra, it can also obtain topological information from the sample surface. This enables correlative analysis of lattice deformation and TERS spectra at specific nanoscale points. We quantified local strain to measure lattice deformation, following a previous method that uses the radius of curvature of the wrinkled structure of monolayer WSe_2_ from STM topography and height data from Figure 1e.^[^ ^11^, ^12^ ^]^ **Figure** **3**a presents the first correlative analysis result, showing the local strain‐dependent properties of the 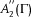 mode. As depicted, the 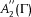 mode in the randomly chosen TERS spectra from Figure 2b shows a linear relationship of phonon frequency and intensity with local strain. To investigate further, we deconvoluted the TERS spectra to identify principal Raman modes, including *E*′(*K*), *E*′(*M*), $A_{1}^{\prime }\left(\Gamma \right)+E^{\prime }\left(\Gamma \right)$, 2LA(*M*), $A_{1}^{\prime }\left(M\right)$, and 3LA(*M*), as shown in Figure 3b, with the resulting deconvoluted spectra displayed in Figure 3c,d. All phonon modes in Figure 3 show similar behavior to the 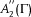 mode. As local strain increases, the phonon frequency shifts linearly toward lower energies, and intensity increases linearly. These principal phonon modes, such as the in‐plane vibrational *E*′(Γ) mode and the LA(*M*) mode, are widely known to exhibit strain‐dependent phonon properties, which clearly coincide with our findings. In particular, the frequency shifts under strain of various phonon modes, including $A_{1}^{\prime }\left(\Gamma \right)$, *E*′(Γ), $A_{1}^{\prime }\left(M\right)$, and 2LA(*M*) are consistent with previous research (e.g., in our study, the 2LA(*M*) mode shifts was −1.94, whereas other reports showed −2.4 and −2.366; for the $A_{1}^{\prime }\left(M\right)$ mode, we obtained −1.628, compared with −1.6 and −1.346 reported elsewhere).^[^ ^6b,j^ ^]^ Notably, our study reveals that the out‐of‐plane vibrational 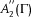 mode also follows a linear relationship with strain. Moreover, the frequency of the out‐of‐plane 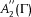 mode is more sensitive to local strain changes than other phonon modes, including in‐plane phonon mode, and is easily distinguishable without deconvolution, as there are no other phonon modes within the 290–320 cm^−1^ range. Consequently, the unique behavior of the 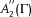 mode suggests it could serve as a new local strain indicator for strain engineering in nanoscale TMD.

### 2.3 Quantum Mechanical Calculations for Mechanical Properties of Mode and Strain‐Induced Selection Rule Relaxation

**Figure 4:**
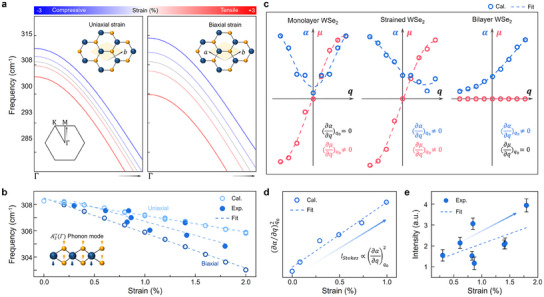
Analysis of 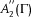 phonon properties with theoretical models. a) Phonon dispersion curve with various applied strain on monolayer WSe_2_. The axes for applied strain have two different cases as depicted in the top of each plot with uniaxial strain and biaxial strain, respectively. b) Coplot of linear dependence of 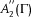 mode derived from theoretical calculation and experimental results. c) Polarizability (α) and dipole moment (μ) change of 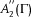 mode with atomic displacement changes for monolayer WSe_2_. d) The linear dependence on strain of strain of the square of first‐order derivative of polarizability change at *q*  =  0. Because the intensity of Stokes Raman scattered light is proportional to the square term, it also implies a linear dependence of the signal intensity on strain. e) The experimental results for linear dependence on strain of 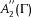 mode intensity. The colored circles, empty circles, and dashed lines indicate the experimental results, the calculated value of physical properties, and the fitted curves, respectively.

Quantum mechanical calculations offer a framework for describing physical properties by simulating actual phenomena without dependence on empirical parameters. The applicability of density functional perturbation theory (DFPT) in providing detailed analyses of phonon variations in deformed atomic lattices has been extensively demonstrated.^[^ ^13^ ^]^ Although previous studies have investigated the strain‐dependent phonon properties using DFPT‐based approaches, none have specifically examined the 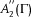 mode through theoretical simulations. This is largely because, in conventional confocal Raman spectroscopy, the Raman‐inactive 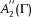 mode is not detected. However, since we have empirically analyzed the behavior of the 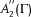 mode using TERS, we now perform DFPT‐based theoretical calculations to verify our experimental findings, investigate the mechanical properties of the 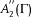 mode, and examine in detail how strain induces relaxation of the Raman selection rule. First, we calculated the phonon dispersion curves of monolayer WSe_2_ under both uniaxial and biaxial strain conditions, with strain values varying from +3% (tensile strain) to −3% (compressive strain). The applied strain directions were aligned based on the primitive vectors of the unit cell. The entire phonon dispersion curves for all three cases, including two uniaxial cases and one biaxial case, are shown in Figure S7 (Supporting Information) which can clearly describe the ideal phonon behaviors in the momentum spaces. **Figure** **4**a provides an enlarged view of the 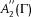 phonon branch near 310 cm^−1^ ranges of dispersion curves in Figure S7 (Supporting Information), which originate from the Brillouin zone center, Γ point. As illustrated, the 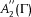 mode exhibits a linear dependence of phonon frequency on applied strain across the entire strain variations. The frequency shift under strain was similar for both uniaxial cases (*a* and *b*), while the biaxial case displayed a relatively larger shift.

To examine the strain‐dependent phonon properties precisely, we conducted dense calculations of the 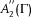 phonon frequency from 0% to 2% strain in 0.2% increments for both uniaxial and biaxial directions. Figure 4b shows these detailed calculated results alongside the experimental TERS spectra, where the slope of the experimental fit lies between the slopes of the uniaxial and biaxial cases, suggesting that strain in wrinkled monolayer WSe_2_ is applied asymmetrically in biaxial directions. Furthermore, this analysis enables us to first derive the Grüneisen parameter, a dimensionless thermodynamic parameter, of the 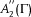 mode that quantifies the degree of energy or frequency change due to strain. This Grüneisen parameter is crucial for using the 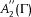 mode as a new local strain indicator, since it represents the sensitivity of phonon frequency to applied strain. According to previous studies, the Grüneisen parameter for the 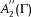 phonon, 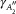, can be derived using the following equations.^[^ ^6^, ^11^ ^]^

(1)

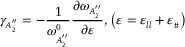



(2)

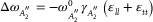

where ε represents the hydrostatic component of strain, *l* is the longitudinal direction, parallel to the strain, and *t* is the transverse direction, as shown in Figure S8 (Supporting Information). Given our local strain calculations are approximated for uniaxial strain, we set ε  = ε_*ll*_, matching the uniaxial cases. Additionally, to calculate 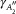 for the biaxial case, a correction factor of 2.366 was applied to account for angle differences between the strain axis and WSe_2_ primitive vectors. Consequently, we obtained Grüneisen parameters of 0.36 and 0.5 from the calculated and experimental results, respectively, which are compatible with each other; minor discrepancies likely arise from approximations in local strain calculations (See Supporting Information for further details).

In the final section, we explore the origin of the anomalous 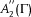 mode appearance, which is typically unobservable in monolayer TMD via conventional Raman spectroscopy due to its Raman‐inactive and IR‐active nature. This relaxation of the Raman selection rule can be approached from both classical and quantum mechanical perspectives. Classically, when atomic vibrations are perturbed by an external electric field, the time‐dependent dipole moment, μ(*t*), can be expressed as follows, where α_0_, *E*_0_, ω_0_, *q*, and ω_*q*_ represent the polarizability at equilibrium state, external electric filed amplitude, external electric field frequency, atomic displacement, and vibrational mode frequency, respectively:^[^ ^14^ ^]^

(3) $$\begin{array}{ccc} & & \mu \left(t\right)=\alpha_{0}\cdot E_{0}\cdot \cos2\pi \left(\omega_{0}t\right)\\ & & +\frac{1}{2}{\left(\partial \alpha /\partial q\right)}_{q_{0}}q_{0}\cdot E_{0}\cdot \left[\cos2\pi \left\{\left(\omega_{0}-\omega_{q}\right)t\right\}+\cos2\pi \left\{\left(\omega_{0}+\omega_{q}\right)t\right\}\right] \end{array}$$

In this equation, the first term represents the Rayleigh scattering process, while the second term with a square bracket denotes the Raman scattering process, split into Stokes and anti‐Stokes scattering components. When ${\left(\partial \alpha /\partial q\right)}_{q_{0}}$, the derivative of electronic polarizability at the equilibrium state, becomes zero the Raman scattering term vanishes, leaving only the Rayleigh scattering process. To apply this classical Raman selection rule to our phenomena, we calculated polarizability and dipole moment changes during the 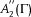 mode, shown in Figure S9 (Supporting Information). By imaging the electrostatic potential (ESP) of monolayer, strained monolayer, and bilayer WSe_2_, we observed polarizability changes during the 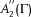 vibration. The enclosed isovolume of ESP indicates the volume of electrons with the same existence probability, proportional to the volume of polarizability ellipsoid, used to identify polarizability variations in atomic vibrations.^[^ ^15^ ^]^ Note that, to monitor the polarizability changes on 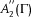 vibrational mode, the enclosed volume in ESP maps could be utilized. Similarly, we marked the Mulliken charge population on the ESP map in Figure S9 (Supporting Information), to derive the trend of dipole moment change.

Figure 4c shows changes in polarizability and dipole moment during the 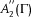 mode vibration, allowing us to determine whether the derivative of polarizability at equilibrium is zero for three distinct cases. As illustrated, the polarizability derivative ${\left(\partial \alpha /\partial q\right)}_{q_{0}}$ for monolayer and bilayer WSe_2_ exhibits zero and non‐zero values, respectively, as expected from point group theory and irreducible representations. However, in strained monolayer WSe_2_, the polarizability derivative becomes non‐zero, indicating that structural deformation due to strain relaxes the Raman selection rule. In other words, the strain‐induced symmetry breaking leads to a non‐zero probability of observing the 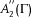 mode via Raman scattering process. While this probability is positive, it remains too low to be detected by conventional methods due to the limited Raman scattering cross‐section, which is consistent with research mentioned above. Here, the strong electromagnetic field enhancement by the gap plasmon near the TERS tip apex allowed us to observe and study this anomalous phonon appearance and softening, as shown in Figure 2.

Additionally, the anomalous appearance of IR‐active vibrational mode through TERS can be explained from a quantum mechanical perspective. When vibrational level transitions occur due to an electric field and field gradient, they are described by a perturbation Hamiltonian that includes dipole–dipole, dipole‐quadrupole, and quadrupole–quadrupole polarizabilities.^[^ ^16^ ^]^ Since the first term is associated with Raman scattering and the second and third terms relate to IR absorption in the visible range, activating dipole‐quadrupole or quadrupole‐quadrupole interactions is essential to observe IR‐active vibrational modes.^[^ ^17^ ^]^ Observing these modes with far‐field vibrational spectroscopy is challenging due to the weak electric field gradient. However, during measuring tip‐enhanced Raman spectra, the strong field gradient at the tip apex enables the excitation and observation of IR‐active vibrational modes through coupling with the dipole‐quadrupole and quadrupole–quadrupole interactions.

Furthermore, we studied the variations in Raman intensity under different strain conditions using a classical physical approach with ESP. The Stokes‐Raman intensity is governed by the square of the polarizability derivate. As the magnitude of this derivative increases, the Stokes scattering intensity also increases, expressed as:

(4) $$I_{Stokes}\propto {\left(\partial \alpha /\partial q\right)}_{q_{0}}^{2}\times {\left(\omega_{0}-\omega_{q}\right)}^{4}\times E_{0}^{2}$$

This proportional relationship is applied to strain‐dependent phenomena. To verify this, we obtained polarizability change curves for strained monolayer WSe_2_ under varying strain levels. As shown in Figure 4d, when tensile strain is increased, ${\left(\partial \alpha /\partial q\right)}_{q_{0}}^{2}$ exhibit a linear dependence on strain magnitude and consistently, the experimental results in Figure 4e and Figure S6 (Supporting Information) show the same linearity. All phonon modes, including the 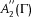 mode, exhibited increased intensity with local strain. Minor deviations in Figure 4e are likely due to substrate‐induced variations in plasmon resonance strength. Our correlative approaches between TERS and DFPT introduce a new strain indicator with significant support through the strong agreement between theoretical calculations and experimental results and the sensitivity of 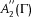 mode presents promising potential for applicability in strain engineering, such as energy harvesting devices, excitonic applications, and SPE.

## 3 Conclusion

In conclusion, we have applied correlative TERS measurements with ambient STM imaging and DFPT‐based calculations to investigate the strain‐dependent phonon properties of monolayer WSe_2_. TERS line trace measurements from the atomic sawtooth gold substrate to the sample interior revealed the collective behavior of various phonon modes, including the out‐of‐plane, Raman‐inactive 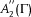 mode, under local strain variations. Local strain calculations based on STM height information quantified and demonstrated the linear relationships between phonon frequency and tensile strain at the nanoscale in strong agreement with calculated phonon dispersion curves. Based on these findings, we derived the Grüneisen parameter for the 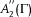 mode, 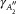, as 0.36 and 0.5 using both theoretical and practical approaches. Furthermore, we examined the relaxation of the Raman selection rule for the 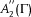 mode via DFPT simulations, confirming strain‐dependent polarizability derivatives through ESP maps that linearly correlate with polarizability ellipsoid changes. This analysis proved the strain‐induced relaxation of the Raman selection rule through the local point symmetry breaking and non‐zero polarizability derivatives, with strong field enhancement from the TERS nanoprobe greatly amplifying the probability of Raman scattering. Our findings and correlative analyses of the 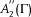 mode establishes it as a sensitive and distinguishable strain indicator for strain engineering in deformed monolayer WSe_2_.

## Conflict of Interest

The authors declare no conflict of interest.

## Supporting information



Supporting Information

## Data Availability

The data that support the findings of this study are available from the corresponding author upon reasonable request.
