# Supplementary material for: Anomalous Phonon Softening with Inherent Strain in Wrinkled Monolayer WSe2
Source: Adv Mater. 2025 Apr 10;37(35):2419414. doi: 10.1002/adma.202419414 (PMC12412005; doi:10.1002/adma.202419414)
Supplement: Supplementary file 1 — Supporting Information [file ADMA-37-2419414-s001.docx]

**Supporting Information**

Anomalous phonon softening with inherent strain from wrinkled monolayer WSe_2_

*Dong Hyeon Kim^a^, Jaekak Yoo^a^, Hyeong Chan Suh^a^, Yo Seob Won^c^, Sung Hyuk Kim^a^, Dong-Joon Yi^b^, Byeong Geun Jeong^c^, Chanwoo Lee^c^, Dongki Lee^d^, Ki Kang Kim^c^, Seung Mi Lee^e^, Eui Kwan Koh^f^, and Mun Seok Jeong^a,^**

^a^Department of Physics, Hanyang University, Seoul 04763, Republic of Korea

^b^Department of Electronic Engineering, Hanyang University, Seoul 04763, Republic of Korea

^c^Department of Energy Science, Sungkyunkwan University, Suwon 16419, Republic of Korea

^d^Department of Nanotechnology and Advanced Materials Engineering, Sejong University, Seoul 05006, Korea

^e^Korea Research Institute of Standards and Science, Daejeon 34113, Republic of Korea

^f^Korea Basic Science Institute, Seoul 02855, Republic of Korea

*Address correspondence to [mjeong@hanyang.ac.kr](mailto:mjeong@hanyang.ac.kr)

**Table of contents**

**Experimental details.**

**Figure S1. Optical microscope images of sample.**

**Figure S2. Microscope images of Au substrate.**

**Figure S3. The extreme performance of tip-enhanced Raman spectroscopy.**

**Figure S4. Far-field Raman spectra of wrinkled monolayer WSe_2_.**

**Figure S5. Linearity between the phonon frequency and local strain.**

**Figure S6. Linearity between the phonon intensity and local strain.**

**Figure S7. Phonon dispersion curves with various applied strain on WSe_2_ unit cell.**

**Figure S8. Schematic illustration for calculation of Grüneisen parameter.**

**Figure S9. Representative electrostatic potential (ESP) maps.**

**Figure S10. Finite-difference time domain (FDTD) simulations of the electric field distribution with and without Au tip.**

**Experimental details**

**Synthesis of monolayer WSe_2_ on atomic sawtooth Au substrate**

The wrinkled monolayer WSe_2_ was synthesized by an atmospheric chemical vapor deposition (CVD) process. In order to synthesize the WSe_2_, a liquid precursor solution was prepared with sodium tungstate dihydrate (Na_2_WO_4_·2H_2_O, Sigma-Aldrich) for W, with 2 wt % in acetylacetone. The liquid precursor solution was coated on the atomic sawtooth Au substrate by using a spin-coater at 2500 rpm for 60 s. Before synthesis, the tube was purged with a 500 sccm of nitrogen gas for 15 minutes at room temperature. To synthesize monolayer WSe_2_, the heating zone 1 was ramped up to 780 °C at a heating rate of 110 °C/min and kept at 780 °C for 10 minutes. For selenization, the temperature of heating zone 2 was increased to 385 °C at a heating rate of 55 °C/min and kept at 385 °C for 10 minutes. The Se vapor was then transported to the sample with 600 sccm N_2_ and 5 sccm H_2_ flow stream. After 10 minutes of growth, heating was stopped, and the furnace was opened to cool naturally to room temperature.^[1]^

**TERS nanoprobe fabrication by automated electrochemical etching**

The TERS nanoprobe was fabricated through automated electrochemical etching process. A gold wire (diameter of 250 μm, purity of 99.95 %, Nilaco) served as the anode and was connected to a waveform generator. The generator applied a square-wave voltage with range between -250 mV to 2.417 V, with a frequency and duty cycle of 300 Hz and 77 %, respectively. A ring-shaped platinum wire (diameter of 200 μm, purity of 99.98 %, Nilaco), used for the cathode in the etching process, was immersed in a potassium chloride-based etchant. The fabricated TERS nanoprobe was rinsed with DI water.^[2]^

**STM-based TERS measurements**

To investigate the phonon properties on strain at nanoscale, we conducted STM-based TERS to measure the plasmon-enhanced Raman spectra of monolayer WSe_2_ on an atomic sawtooth gold substrate, which can incorporate the extreme conditions. In particular, the nanocavity formed between the gold tip and the atomic sawtooth gold substrate gives rise to a gap-mode plasmon, which strongly enhances the local electric field and thereby amplifies Raman scattered signal, including $A_{2}^{''}(\Gamma)$ mode.

The TERS system (NTEGRA Spectra, NT-MDT) is composed of both scanning tunneling microscopy (STM) and a confocal Raman scattering system. Preceding the TERS scanning process, STM imaging was employed to scrutinize the edge interface of the wrinkled WSe_2_ monolayer. The STM images were acquired under specific scanning conditions, with a tunneling current of approximately 0.1 nA and a bias voltage of 0.15 V, within an ambient environment. The TERS scanning procedure utilized an excitation laser with a wavelength of 632.8 nm and an objective lens possessing a numerical aperture (NA) of 0.7 (Mitutoyo). Multispectral TERS spectra were acquired through a spectrometer featuring 1800 grooves/mm grating, blazed at 500 nm, and an EMCCD (Andor) cooled to a temperature of -65 °C. The same gold nanoprobe was used for all STM and TERS measurements.

To prevent tip drift issues during measurements, the angle between the gold nanoprobe and monolayer WSe_2_ was precisely controlled. The polarization of the incident laser with 632.8 nm wavelength was also accurately aligned with the orientation of the gold nanoprobe to fully excite localized surface plasmon resonance (LSPR), achieved by rotating the half-wave plate. The piezo transducer of the sample stage enabled precise nano-positioning of the gold nanoprobe as the tunneling current changes between the gold tip and substrate.^[3]^

**Theoretical calculation of diverse phonon properties**

To determine the vibrational frequencies and phonon dispersion of tungsten diselenide (WSe_2_), the density functional perturbation theory (DFPT) was calculated using the plane-wave method (CASTEP) implemented in the BIOVIA Materials Studio platform. Pristine WSe_2_ was modeled and geometry optimized with local density approximation (LDA) functional. Because perturbation calculation requires a high level of structural stabilization, the cutoff of 830 eV, force convergence tolerance of 0.01 eV/Å, and self-consistent field (SCF) tolerance of 1.0 x 10^-10^ eV/atom were implemented. Monkhorst-Pack grid was set to have an actual spacing of 0.02 Å^-1^ to achieve sufficient computational precision. The norm-conserving pseudopotential and Koelling-Harmon relativistic treatment were used for the calculation process. After that, we augmented the inter-layer distance vacuum slab by 20 Å, representing a fully isolated layered structure under the periodic boundary condition. The proposed optimized pristine monolayer of WSe_2_ is in good agreement with previous reports (our result: a = 3.268 Å, d_W-Se_ = 2.52 Å, d_Se-Se_ = 3.34 Å; reference: a = 3.275 Å, d_W-Se_ = 2.51Å, d_Se-Se_ = 3.34 Å).^[4]^

To investigate the strain-induced Raman peak shift, we calculated the linear response with interpolation method with the 1 %, 2 %, and 3 % of tensile- and compressive-strained WSe_2_ monolayer after geometry optimization. The negative acoustic phonon energy near $\Gamma$ position in phonon dispersion represents the over 3 % of strain occurs over deformation of lattice. To explain the degree of strain effect precisely, we calculated the tensile-strained structures from 0 % to 2 % with 0.2 % step for both biaxial and uniaxial direction. In the case of uniaxial strained structure, we considered for both *a* and *b* direction, which are unique component of lattice plane direction.

To further investigate strain-induced Raman intensity variations, the polarizability and dipole moments of both the pristine and uniaxially strained (1 % along the *b* direction) WSe_2_ structures were calculated. Using the DMOL3 code in BIOVIA Materials Studio, we performed calculations with double numeric atomic orbitals and polarization basis sets, considering full-electron treatment with relativistic effects for the core. The LDA functional was used for consistency with previous calculations. Vibrational calculations were first performed to identify the out-of-plane vibrational modes. From these modes, a total of 24 structural frames were extracted. For each frame, we calculated the electrostatic potential of the total electron density and Mulliken charge population. Polarizability was determined by analyzing the differences in electrostatic volume between each frame and the origin frame, which corresponds to the initial structure before the vibrational motion. Similarly, dipole moments were calculated by evaluating the changes in atomic positions and charge population between each frame and the origin frame.

**Quantifying local strain on wrinkled monolayer WSe_2_**

The synthesized monolayer WSe_2_ on atomic sawtooth Au substrate is subjected to tensile strain as it bends locally to conform to the substrate geometry. To investigate the correlation between phonon properties and local strain, we quantified the local strain on WSe_2_ wrinkle using an isogeometric analysis method based on the classical elasticity theory. The step-like structure of atomic sawtooth Au substrate primarily applies uniaxial strain to the wrinkled monolayer TMD, which is analogous to bending TMD materials on a flexible substrate. In particular, the direct growth of monolayer WSe_2_ on Au substrate allows us to regard the edges have been clamped. Under these assumptions, the equations to determine the local strain on monolayer WSe_2_ can be derived as follows:

$$\varepsilon_{xx}\left( x \right)=-\tau/{[2\left( {\partial^{2}h}/{\partial x^{2}} \right)]}$$

where $\varepsilon_{xx}$ is the applied strain, $\tau$ is the thickness of monolayer WSe_2_, and $h$ is the deflection. In addition, if the deflection is smaller than the thickness of material, the equation can be expressed as below:

$$\varepsilon_{xx}\left( x \right)=\tau/{2R}$$

where $R$ denotes the radius of curvature.^[5]^

**Finite-Difference Time-Domain (FDTD) simulations**

FDTD simulations were performed using the open-source software Meep (version 1.29.0) to investigate the optical field distribution in proximity to a metallic nanostructure.^[6]^ A monochromatic light source with a wavelength of 632.8 nm was employed for excitation, with its linear polarization oriented parallel to the tip axis. To maintain computational efficiency while accurately representing the key features of the experimental setup, the focused Gaussian beam was approximated as a plane wave.

To systematically assess the effects of surface topography and tip-induced field enhancement, four distinct configurations were considered: (i) a structured Au substrate with a sawtooth pattern characterized by a periodicity of 50 nm and a height of 15 nm, (ii) a flat Au substrate, (iii) a structured Au substrate with an Au tip, and (iv) a flat Au substrate with an Au tip. In configurations incorporating a tip, a Au tip with an apex radius of 10 nm was positioned 3 nm above the substrate to maintain a consistent tip-substrate separation.

To closely replicate experimental conditions, a single plane wave source was introduced at incidence angles of 0° and 60° relative to the substrate. A Discrete Fourier Transform monitor was employed to capture the electromagnetic field distribution within the simulation domain, facilitating a quantitative evaluation of localized field enhancement. This analysis was used to elucidate the impact of surface structuring and the presence of a tip on field enhancement characteristics.

To ensure the validity of the simulation approach, computational parameters were carefully selected to align with experimentally relevant conditions. The results provide critical insights into the underlying mechanisms governing nanoscale optical interactions, contributing to a deeper understanding of field enhancement phenomena near metallic nanostructures.


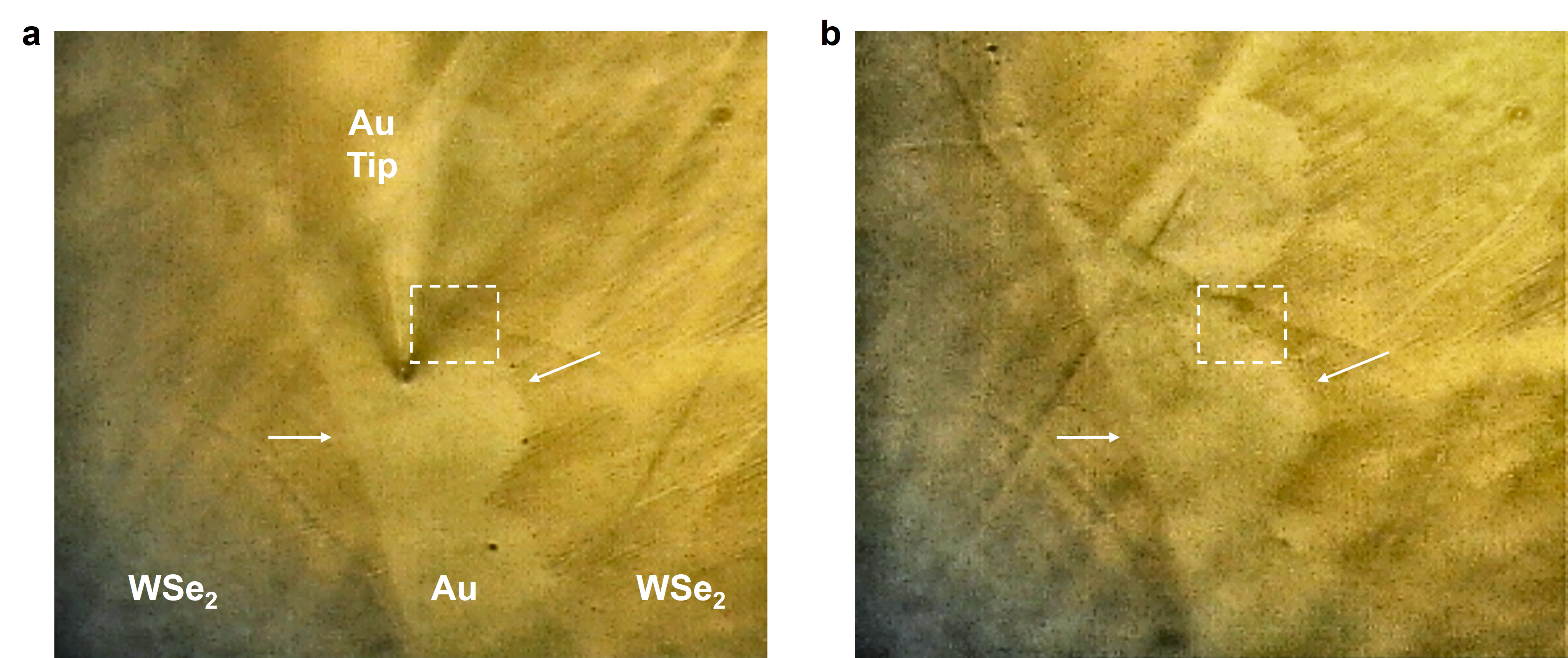


**Figure S1. Optical microscope images of sample.** The optical microscope images of monolayer WSe_2_ synthesized on atomic sawtooth gold substrate (a) with gold tip and (b) without gold tip. The white dashed rectangles indicate the STM scanned area, and the white arrows indicate the boundary between the gold substrate and monolayer WSe_2_.

**
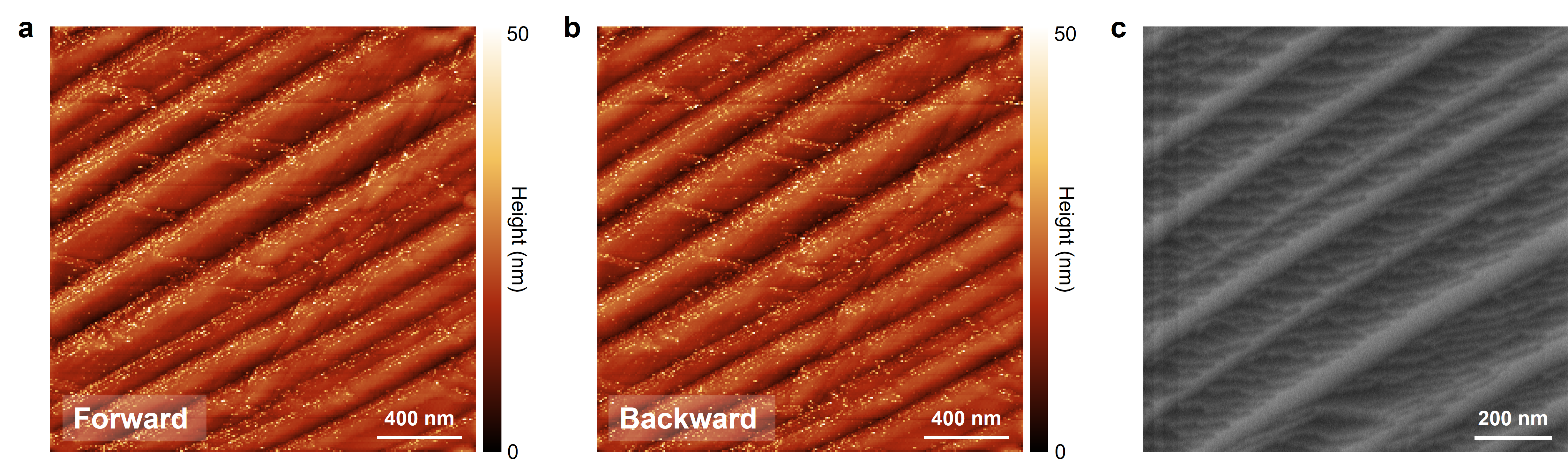
**

**Figure S2. Microscope images of Au substrate.** The scanning tunneling microscope images of atomic sawtooth Au substrate for (a) forward scan direction and (b) backward scan direction. (c) The scanning electron microscope image of atomic sawtooth Au substrate. The nanoscale steps consist of atomic sawtooth steps.

**
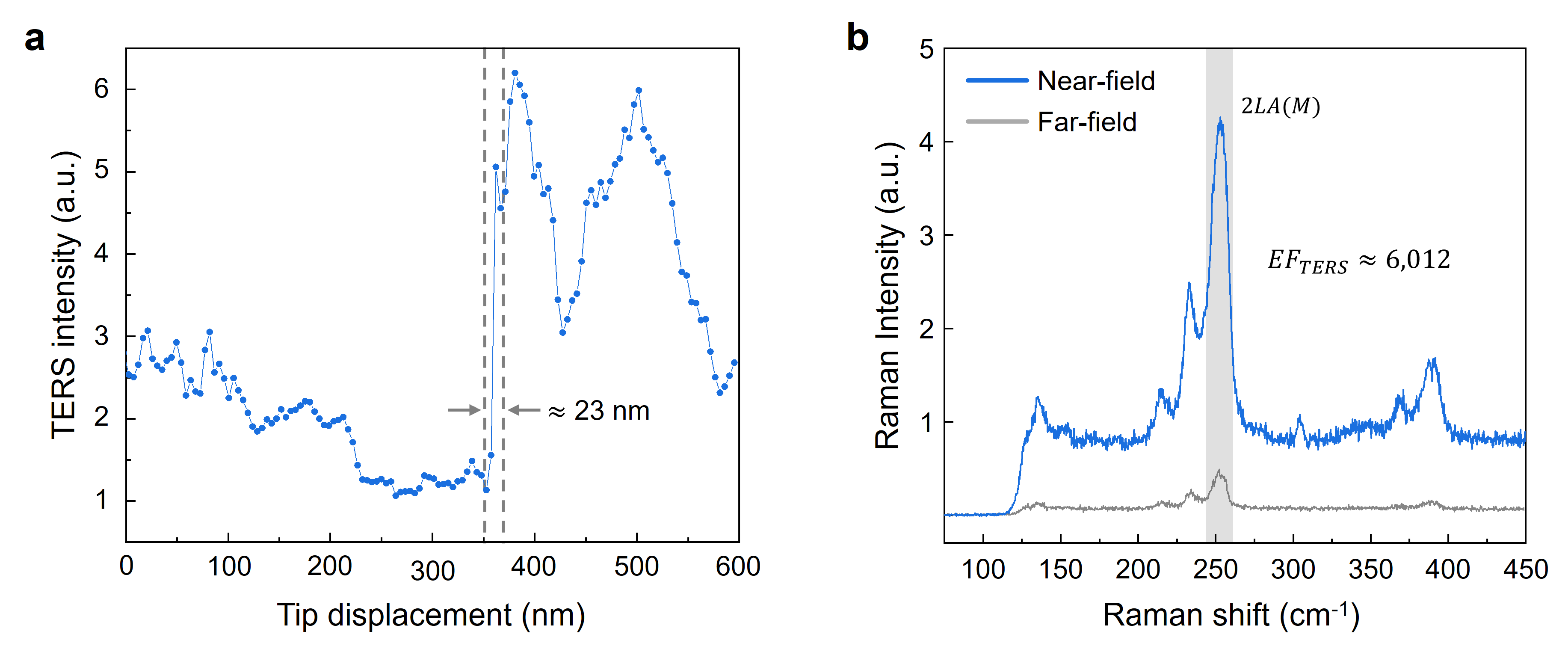
**

**Figure S3. The extreme performance of tip-enhanced Raman spectroscopy.** (a) Tip-enhanced Raman scattered signal intensity profile of monolayer WSe_2_ along the tip displacement. The dashed grey lines indicate the spatial resolution of this system that is around 23 nm. (b) Near-field (blue curve) and far-field (grey curve) Raman spectra of monolayer WSe_2_ which were measured at the same position. The enhancement factor of TERS is around 6,012.

**
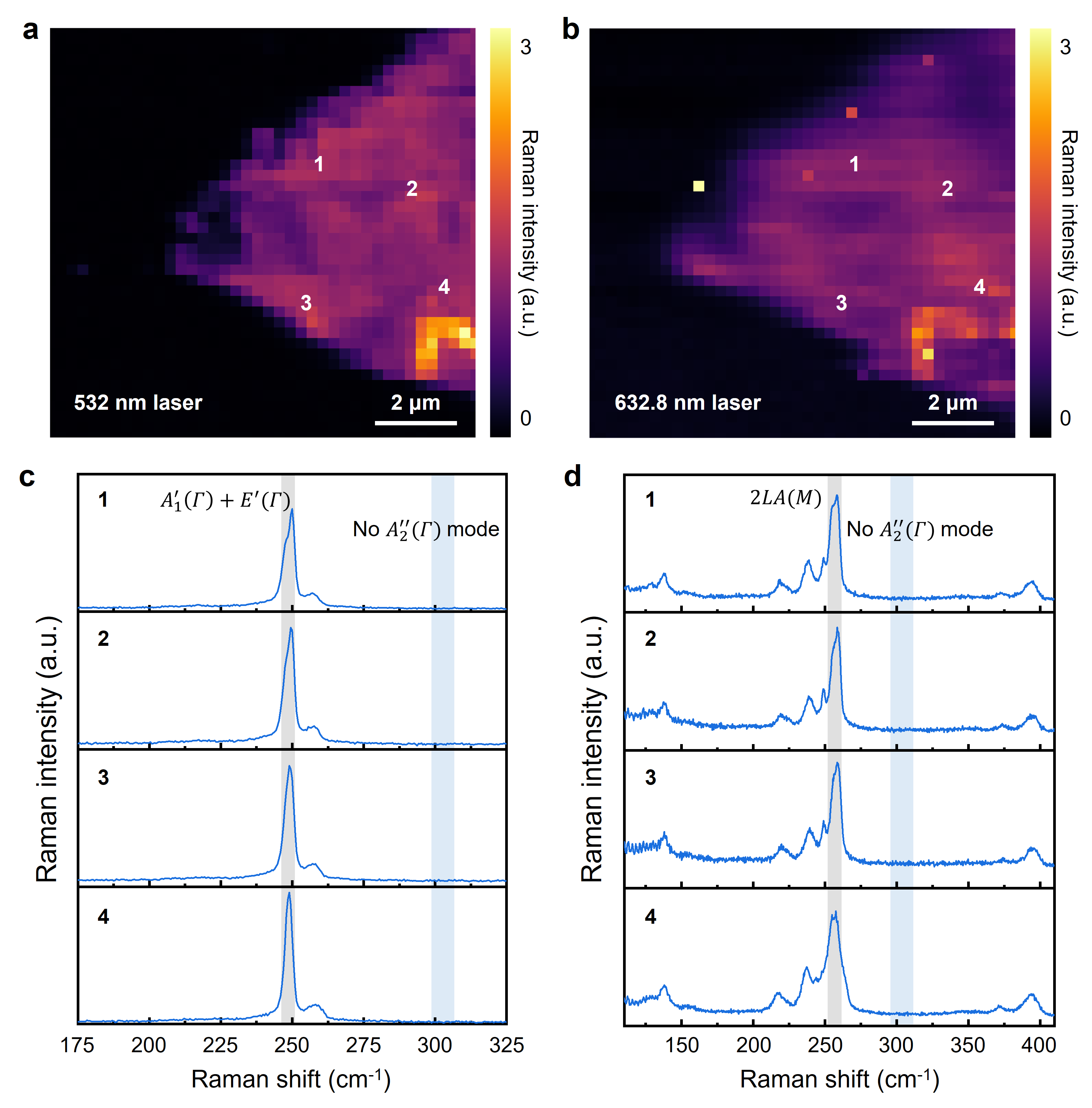
**

**Figure S4. Far-field Raman spectra of wrinkled monolayer WSe_2_.** (a, b) The representative far-field Raman intensity map of wrinkled monolayer WSe_2_ on Au substrate with two excitation laser sources. (c, d) The representative far-field Raman spectra of four random points marked in (a, b). The absence of $A_{2}^{''}(\Gamma)$ mode confirms that the wrinkled WSe_2_ sample is monolayer.

**
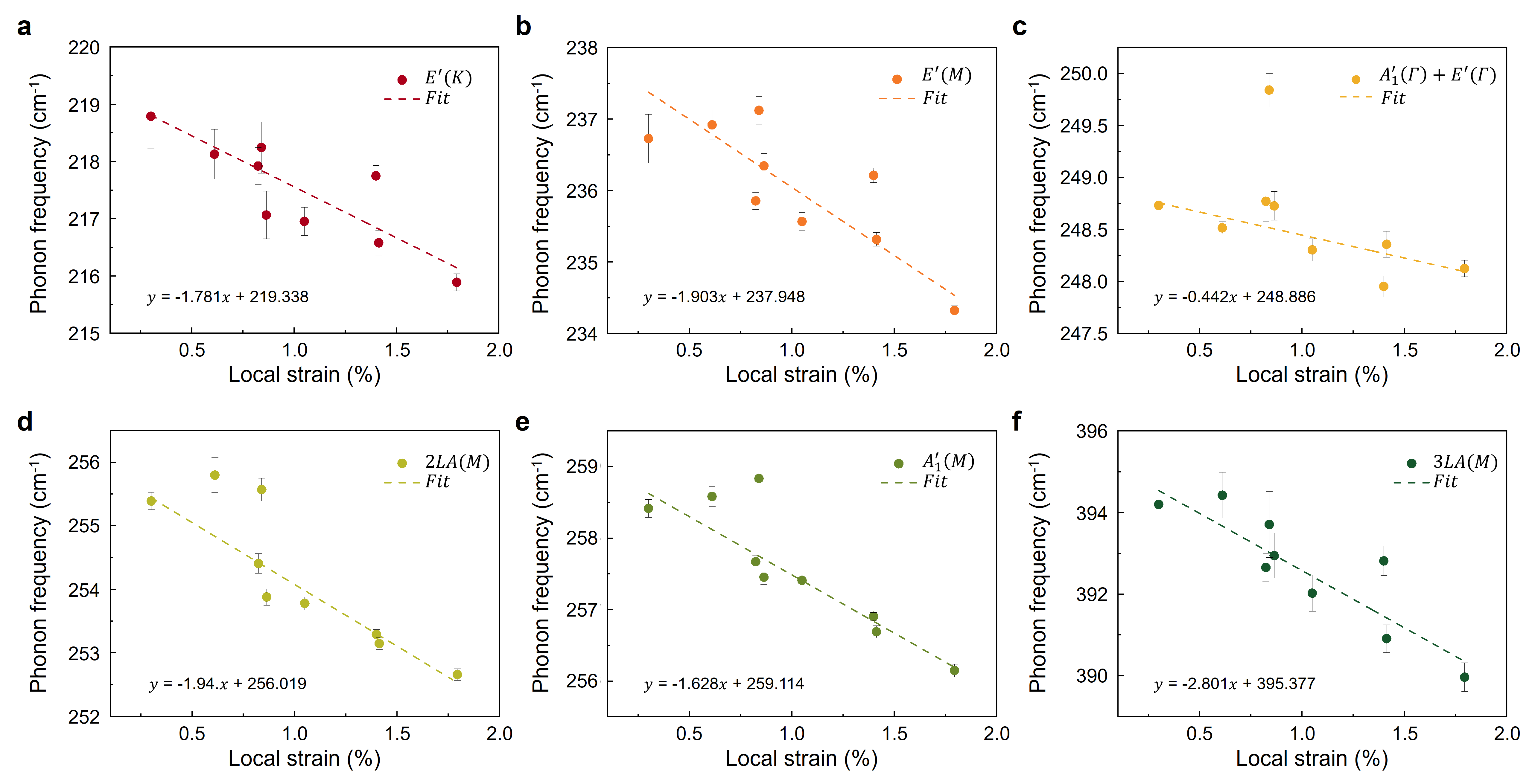
**

**Figure S5. Linearity between the phonon frequency and local strain.** The enlarged linearity between the phonon frequency and local strain in Figure 3c in manuscript with error bar for deconvolution process. (a-f) indicate the linearity of phonon $E^{'}(K)$, $E^{'}(M)$, $A_{1}^{'}\left( \Gamma\right)+E^{'}(\Gamma)$, $2LA(M)$, $A_{1}^{'}\left( M \right)$, $3LA(M)$, respectively. All the phonons show softening as the local strain increases.


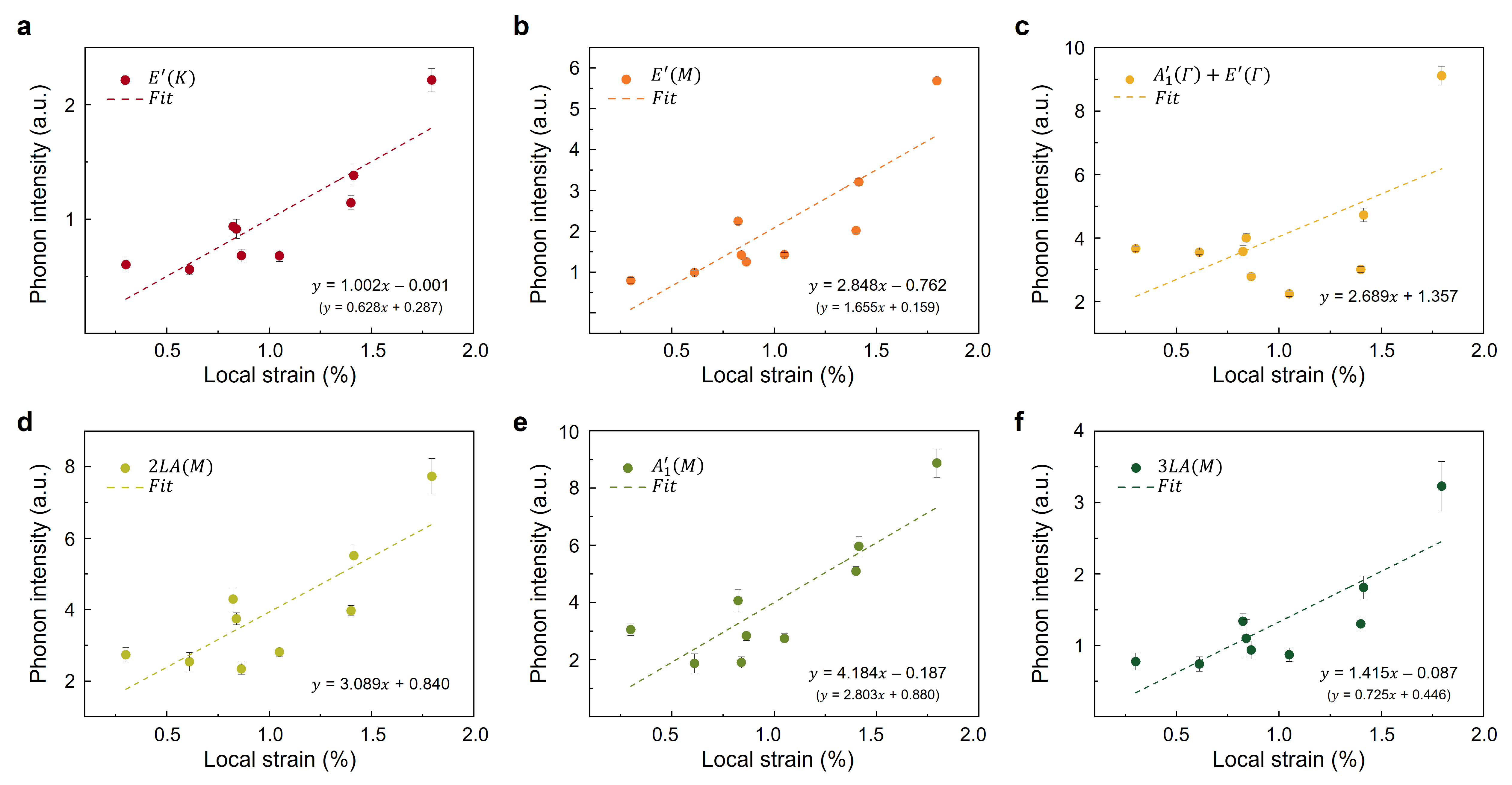


**Figure S6. Linearity between the phonon intensity and local strain.** The enlarged linearity between the phonon intensity and local strain in Figure 3d in manuscript with error bar for deconvolution process. (a-f) indicate the linearity of phonon $E^{'}(K)$, $E^{'}(M)$, $A_{1}^{'}\left( \Gamma\right)+E^{'}(\Gamma)$, $2LA(M)$, $A_{1}^{'}\left( M \right)$, $3LA(M)$, respectively. All the phonons show intensity enhancement as the local strain increases. Negative intercepts in the phonon intensity data may be due to outliers. In contrast, the linear fit equations (shown in round brackets), derived after excluding these outliers, yield positive intercepts. These unusual phonon intensities may be influenced by variations in local plasmon resonance strengths.

**
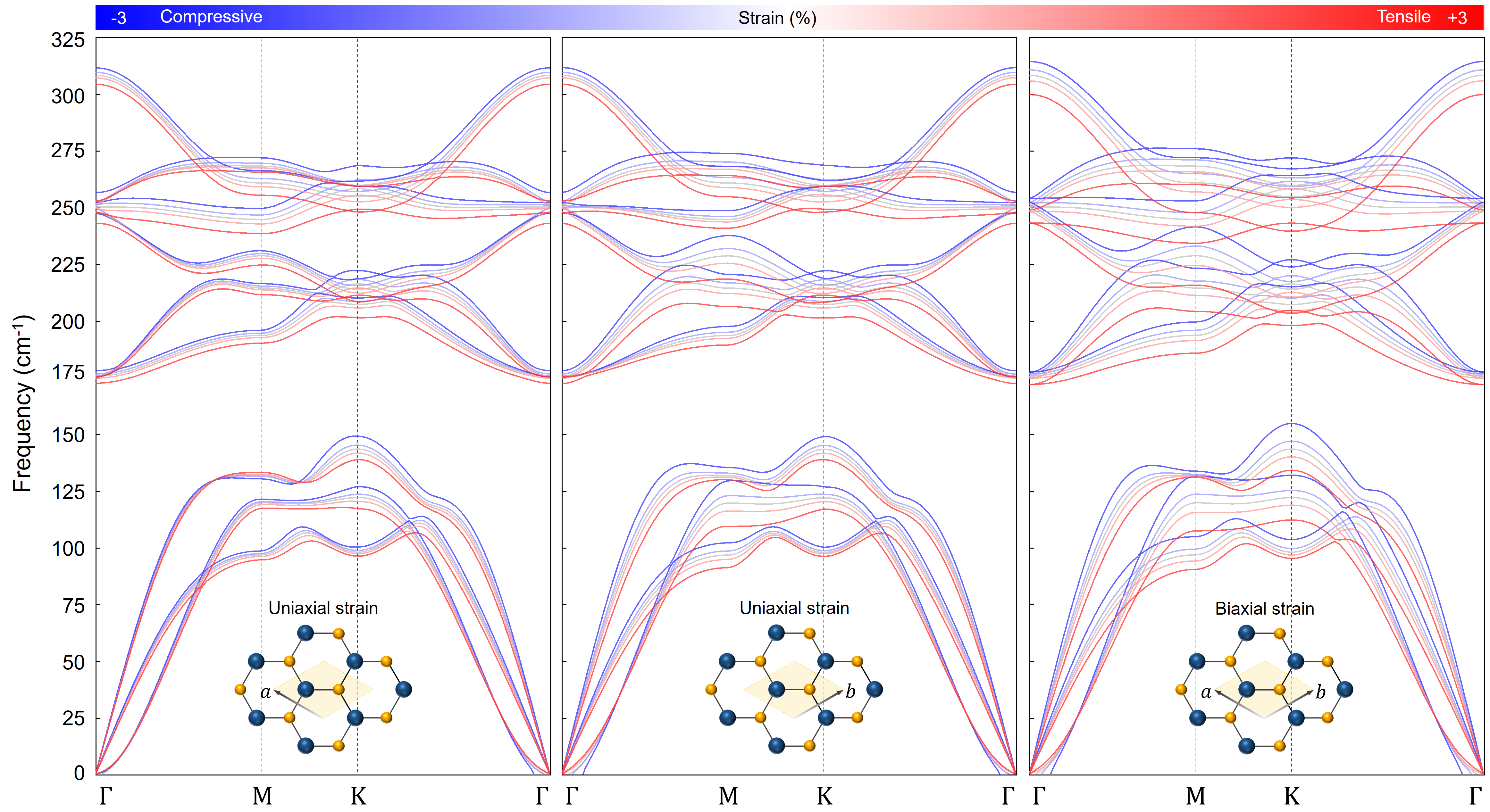
**

**Figure S7. Phonon dispersion curves with various applied strain on WSe_2_ unit cell.** The axes for applied strain have three different cases as depicted in the bottom of each plot with uniaxial strain a, b, and biaxial strain.

**
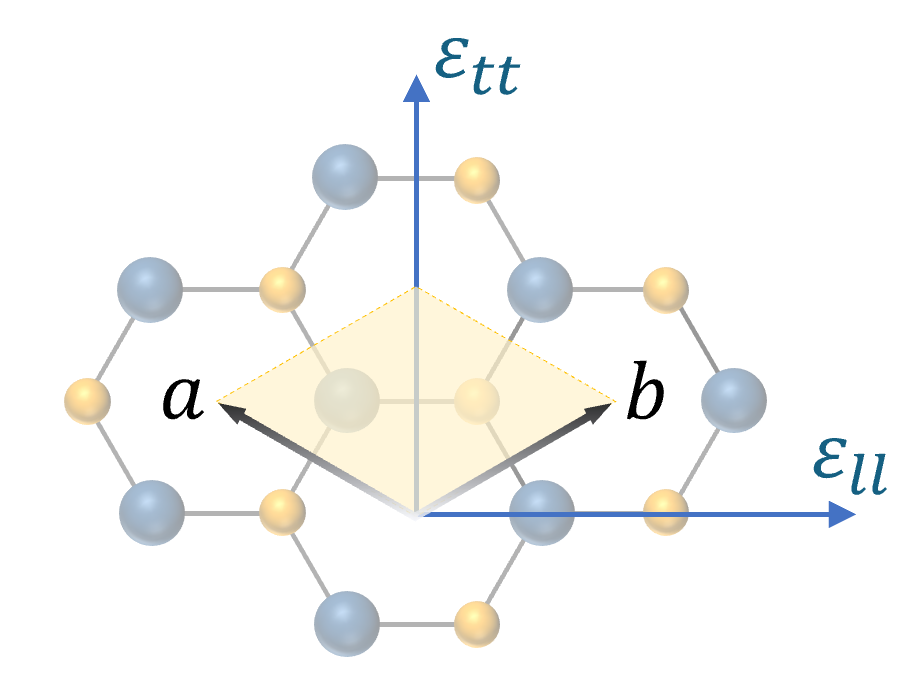
**

**Figure S8. Schematic illustration for calculation of Grüneisen parameter.** The parameters $\varepsilon$, $l$, and $t$ indicates the hydrostatic component of strain, the longitudinal direction (parallel to strain) and the transverse direction (perpendicular to strain), respectively.

**The discrepancy of Grüneisen parameters of** $\boldsymbol{A}_{\boldsymbol{2}}^{\boldsymbol{''}}\left( \boldsymbol{\Gamma} \right)$**mode of monolayer WSe_2_**

In this study, we experimentally and theoretically derived the Grüneisen parameter of the Raman-inactive $A_{2}^{''}\left( \Gamma\right)$mode, obtaining values of 0.5 and 0.36, respectively. When comparing experimentally and theoretically determined Grüneisen parameters, discrepancies can arise because strain is handled differently in each approach. Experimentally, local strain is often calculated from topographic data acquired *via* scanning probe microscopy (SPM), which requires assuming that the sample edges are clamped. In contrast, density functional theory (DFT) calculations of phonon frequencies under strain typically use free boundary conditions, meaning the sample is unconstrained at the edges.^[4, 7]^ As a result, the effective strain in the sample may differ between experimental and theoretical scenarios.

Since the Grüneisen parameter essentially corresponds to the slope of the phonon frequency with respect to strain, these boundary-condition differences can significantly affect the derived values. Moreover, in real experiments, the strain applied to the sample is rarely purely uniaxial or perfectly uniform along both axes, leading to anisotropic strain effects that can introduce additional error in experimentally extracted values. Thus, these factors can explain why the derived Grüneisen parameters differ between the two approaches.


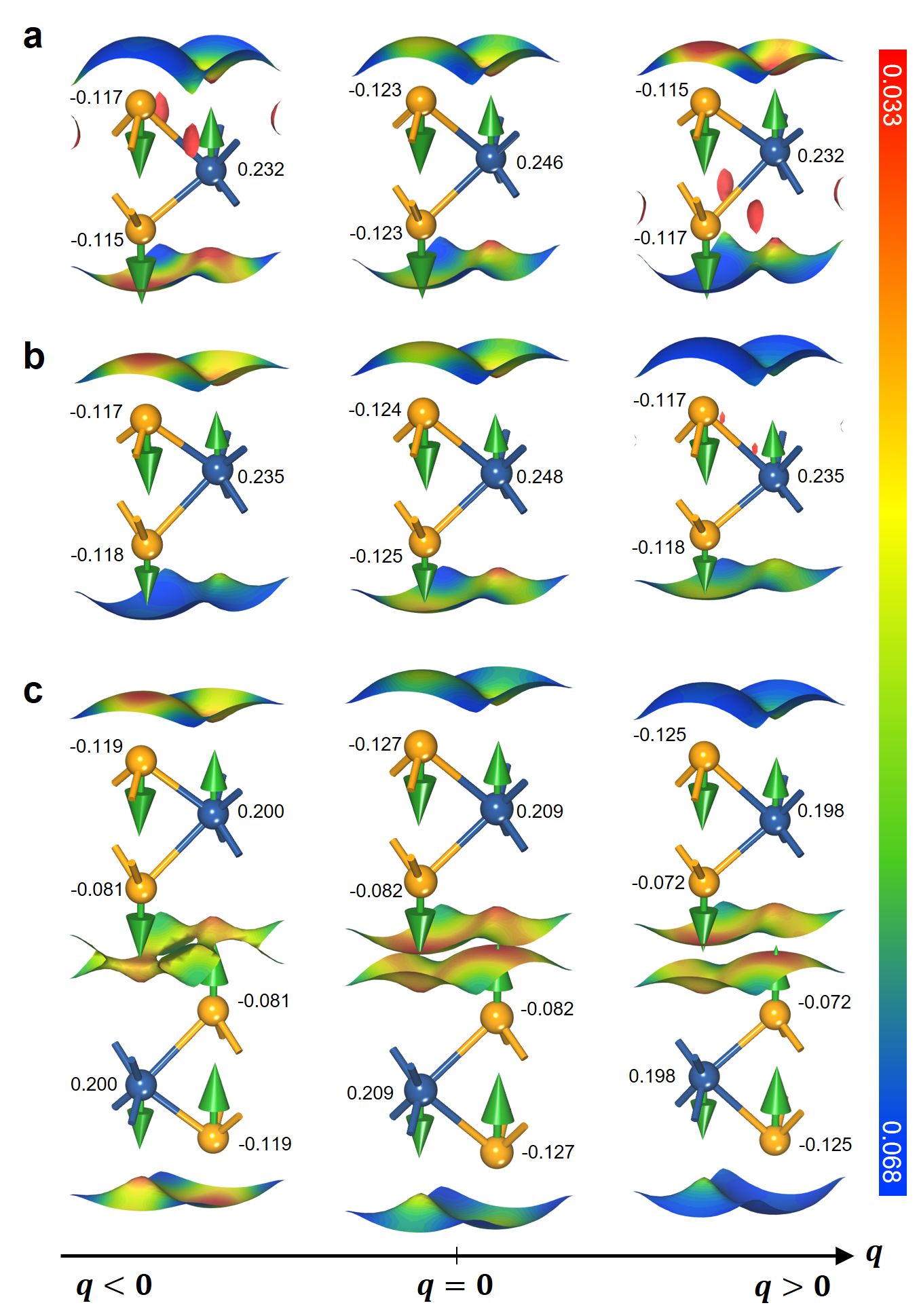


**Figure S9. Representative electrostatic potential (ESP) maps.** ESP on the WSe_2_ (a) monolayer (b) strain induced monolayer (c) bilayer for $A_{2}^{''}\left( \Gamma\right)$ mode in each $q>0$, $q=0$, $q<0$. Valued in atomic units.

**The relaxation of point group of monolayer WSe_2_ with local strain**

In general, pristine monolayer WSe_2_ belongs to the $D_{3h}$ point group, exhibiting high symmetry. However, significant strain can lower this symmetry. Specifically, depending on the extent, direction, and symmetry of the applied strain, the $D_{3h}$ point group of monolayer WSe₂ can be reduced to $C_{3v}$, $C_{2v}$, $C_{s}$, or $C_{1}$. In the process, the $A_{2}^{''}$ vibrational mode can transform into $A_{1}$, $A^{'}$, or $A$. For instance, under uniaxial strain, the threefold rotational axis and horizontal mirror plane are broken, reducing the point group to $C_{2v}$. Further breaking of the twofold rotational axis and vertical mirror plane can lower it to $C_{s}$ or even $C_{1}$. At each stage of this symmetry relaxation, additional quadratic tensors can alter the Raman activity of the $A_{2}^{''}$ vibrational mode, which is initially IR-active. Because these extreme point group variations occur only at the nanoscale, tip-enhanced Raman spectroscopy (TERS) is required to experimentally probe them *via* strong field enhancement within the nanocavity. Table 1 summarizes the various point groups that emerge under local strain, along with the corresponding vibrational modes and their optical activities.^[8]^

**Table S1. Various point groups and referred out-of-plane vibrational mode of monolayer WSe_2_.**

| **Point group** | **Vibrational mode** | **Linear vector, rotation** | **Quadratic tensor** | **Activity** |
| --- | --- | --- | --- | --- |
| $D_{3h}$ | $A_{2}^{''}$ | $z$ | None | IR-active |
| $C_{3v}$ | $A_{1}$ | $z$ | $x^{2}+y^{2}$, $z^{2}$ | IR-active, Raman-active |
| $C_{2v}$ | $A_{1}$ | $z$ | $x^{2}$, $y^{2}$, $z^{2}$ | IR-active, Raman-active |
| $C_{s}$ | $A^{'}$ | $x$, $y$, $R_{z}$ | $x^{2}$, $y^{2}$, $z^{2}$, $xy$ | IR-active, Raman-active |
| $C_{1}$ | $A$ | - | - | IR-active, Raman-active |


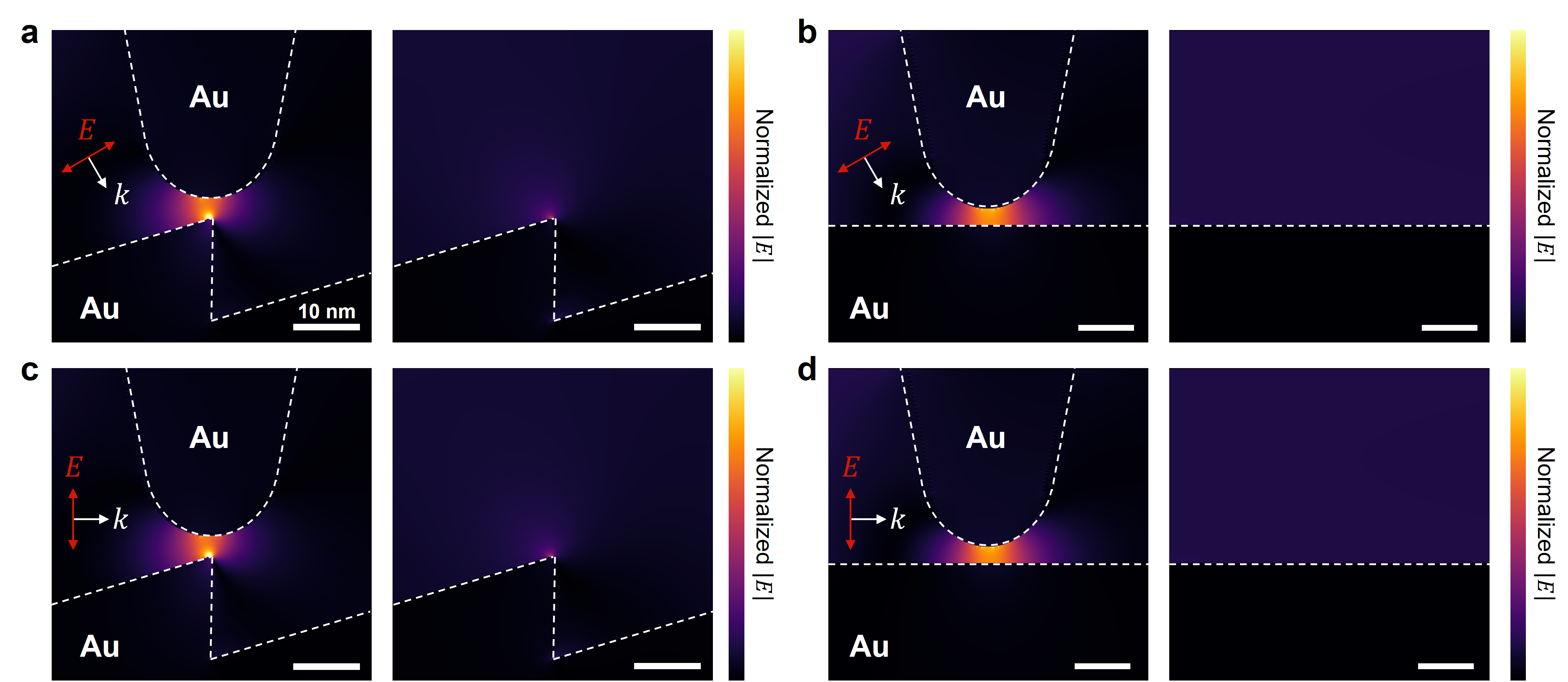


**Figure S10. Finite-difference time domain (FDTD) simulations of the electric field distribution with and without Au tip.** (a, b) Normalized electric-field distribution maps under linearly polarized light oriented along the sawtooth slope, for (a) an atomic sawtooth Au substrate and (b) a flat Au substrate. (c, d) Normalized electric-field distribution maps under linearly polarized light oriented along the tip, for (c) an atomic sawtooth Au substrate and (d) a flat Au substrate. All white scale bars represent 10 nm. When the Au tip approaches the atomic sawtooth Au substrate, the field intensity is enhanced by more than tenfold. The electric field was monitored through the component corresponding to the y axis direction.

**References**

[1] a)S. H. Choi, H.-J. Kim, B. Song, Y. I. Kim, G. Han, H. T. T. Nguyen, H. Ko, S. Boandoh, J. H. Choi, C. S. Oh, H. J. Cho, J. W. Jin, Y. S. Won, B. H. Lee, S. J. Yun, B. G. Shin, H. Y. Jeong, Y.-M. Kim, Y.-K. Han, Y. H. Lee, S. M. Kim, K. K. Kim, *Advanced Materials* **2021**, 33, 2006601; b)H. Ko, S. H. Choi, Y. Park, S. Lee, C. S. Oh, S. Y. Kim, Y. H. Lee, S. M. Kim, F. Ding, K. K. Kim, *Nature Communications* **2024**, 15, 5848.

[2] a)C. Lee, S. T. Kim, B. G. Jeong, S. J. Yun, Y. J. Song, Y. H. Lee, D. J. Park, M. S. Jeong, *Scientific Reports* **2017**, 7, 40810; b)D. H. Kim, C. Lee, B. G. Jeong, S. H. Kim, M. S. Jeong, **2020**, 9, 2989.

[3] a)C. Lee, B. G. Jeong, S. J. Yun, Y. H. Lee, S. M. Lee, M. S. Jeong, *ACS Nano* **2018**, 12, 9982; b)D. H. Kim, C. Lee, S. H. Kim, B. G. Jeong, S. J. Yun, H. C. Suh, D. Lee, K. K. Kim, M. S. Jeong, **2024**, 13, 1069.

[4] a)C. Ataca, H. Şahin, S. Ciraci, *The Journal of Physical Chemistry C* **2012**, 116, 8983; b)H. Nakamura, A. Mohammed, P. Rosenzweig, K. Matsuda, K. Nowakowski, K. Küster, P. Wochner, S. Ibrahimkutty, U. Wedig, H. Hussain, J. Rawle, C. Nicklin, B. Stuhlhofer, G. Cristiani, G. Logvenov, H. Takagi, U. Starke, *Physical Review B* **2020**, 101, 165103.

[5] a)E. M. L. L. D. Landau, *Theory of Elasticity*, Vol. 7, Pergamon Press, **1959**; b)S. W.-K. S. Timoshenko, *Theory of Plates and Shells*, McGraw-Hill, **1959**; c)A. M. Dadgar, D. Scullion, K. Kang, D. Esposito, E. H. Yang, I. P. Herman, M. A. Pimenta, E. J. G. Santos, A. N. Pasupathy, *Chemistry of Materials* **2018**, 30, 5148; d)T. P. Darlington, A. Krayev, V. Venkatesh, R. Saxena, J. W. Kysar, N. J. Borys, D. Jariwala, P. J. Schuck, *The Journal of Chemical Physics* **2020**, 153, 024702; e)S.-S. Wu, T.-X. Huang, X. Xu, Y.-F. Bao, X.-D. Pei, X. Yao, M.-F. Cao, K.-Q. Lin, X. Wang, D. Wang, B. Ren, *ACS Nano* **2022**, 16, 4786.

[6] a)A. F. Oskooi, D. Roundy, M. Ibanescu, P. Bermel, J. D. Joannopoulos, S. G. Johnson, *Computer Physics Communications* **2010**, 181, 687; b)D. Y. Lee, C. Park, J. Choi, Y. Koo, M. Kang, M. S. Jeong, M. B. Raschke, K.-D. Park, *Nature Communications* **2021**, 12, 3465.

[7] a)C.-H. Chang, X. Fan, S.-H. Lin, J.-L. Kuo, *Physical Review B* **2013**, 88, 195420; b)H. Sahin, S. Tongay, S. Horzum, W. Fan, J. Zhou, J. Li, J. Wu, F. M. Peeters, *Physical Review B* **2013**, 87, 165409; c)M. Yagmurcukardes, C. Bacaksiz, E. Unsal, B. Akbali, R. T. Senger, H. Sahin, *Physical Review B* **2018**, 97, 115427.

[8] a)F. A. Cotton, *Chemical Applications of Group Theory*, Wiley, **1991**; b)J. O. D. George F. Koster, Robert G. Wheeler, Hermann Statz, *The Properties of The Thirty-Two Point Groups*, The MIT Press, **1963**; c)G. D. Mildred S. Dresslhaus, Ado Jorio, *Group Theory (Application to the Physics of Condensed Matter)*, Springer Berlin, Heidelberg, **2007**.
